# Supplementary material for: Gluten Conformation at Different Temperatures and Additive Treatments
Source: Foods. 2022 Feb 1;11(3):430. doi: 10.3390/foods11030430 (PMC8834346; doi:10.3390/foods11030430)
Supplement: Supplementary file 1 [file foods-11-00430-s001.zip › foods-1569987-supplymentary.pdf]

**Table S1.** Gluten secondary structure (FTIR spectroscopy) expressed in area (%) as a function of temperature, ascorbic acid (AA), diacetyl tartaric acid ester of monoglycerides (DATEM) and dithiothreitol (DTT)[a].

| Temperature | Additive | Area (%) | | | | |
|---|---|---|---|---|---|---|
| | | **Intermolecular β-sheets/ extended hydrated chains** | **Antiparallel β-sheets, more weakly hydrogen-bonded β-sheets** | **Random coils and α-helices** | **β-turns** | **β-sheets** |
| | Control | $26.4^{f}$ | $24.3^{d}$ | $23.5^{e}$ | $5.6^{b}$ | $8.7^{c}$ |
| 25 °C | AA | $29.5^{a}$ | $24.3^{d}$ | $22.3^{g}$ | $5.3^{b}$ | $7.3^{g}$ |
| | DATEM | $25.5^{h}$ | $25.0^{b}$ | $25.7^{ab}$ | $7.5^{a}$ | $6.5^{i}$ |
| | DTT | $27.1^{e}$ | $25.6^{a}$ | $22.9^{f}$ | $5.6^{b}$ | $7.8^{e}$ |
| 45 °C | AA | $28.9^{b}$ | $24.2^{d}$ | $24.0^{cd}$ | $4.6^{c}$ | $7.6^{f}$ |
| | DATEM | $26.5^{f}$ | $24.3^{d}$ | $23.8^{de}$ | $4.4^{cd}$ | $9.2^{b}$ |
| | DTT | $26.4^{f}$ | $24.2^{d}$ | $23.8^{de}$ | $3.4^{e}$ | $10.4^{a}$ |
| 65 °C | AA | $27.9^{c}$ | $23.8^{e}$ | $25.7^{a}$ | $3.1^{e}$ | $8.2^{d}$ |
| | DATEM | $26.3^{g}$ | $24.9^{c}$ | $25.2^{b}$ | $4.1^{d}$ | $8.3^{d}$ |
| | DTT | $27.5^{d}$ | $23.8^{e}$ | $24.5^{c}$ | $4.8^{c}$ | $7.1^{h}$ |

[a] Mean values (n = 3) within the same column followed by the same superscript letter are not significantly different (Tukey's test $P<0.05$). Treatments: 100 ppm AA, 0.6% DATEM and 25 mM DTT.
